# Fatal Flaw in the Folate Pathway: Methotrexate-Bactrim Interaction, Misleading Levels, and a Cautionary Case

**DOI:** 10.7759/cureus.99984

**Published:** 2025-12-24

**Authors:** Shaivya Pathak

**Affiliations:** 1 Internal Medicine, East Carolina University, Greenville, USA

**Keywords:** drug-drug interaction, methotrexate toxicity, pancytopenia, rheumatoid arthritis, trimethoprim-sulfamethoxazole

## Abstract

Methotrexate (MTX) is one of the most commonly prescribed medications for rheumatoid arthritis and other autoimmune disorders. While therapeutic drug monitoring (TDM) protocols are well-established for high-dose MTX (HD-MTX) in oncology, no such validated protocols exist for low-dose MTX (LD-MTX) therapy - the pharmacokinetics are highly variable, and serum concentrations correlate poorly with clinical toxicity. Among the most dangerous drug interactions is the combination of MTX with trimethoprim-sulfamethoxazole (TMP-SMX), which creates synergistic toxicity. This case details a rapid, fatal MTX toxicity event in a patient on stable LD therapy who received a short course of TMP-SMX for cellulitis. Severe pancytopenia and hemorrhagic oral mucositis developed despite a serum MTX concentration of 0.07 μmol/L - well below the commonly accepted toxic threshold. This case underscores that clinicians must prioritize prompt recognition of clinical symptoms over laboratory quantification when risk factors for drug interaction are present.

## Introduction

Methotrexate (MTX), a folic acid antagonist and cell cycle-specific antimetabolite, is one of the most commonly prescribed medications for rheumatoid arthritis and other autoimmune disorders. While historically used in high doses (HD-MTX, ≥500 mg/m²) for hematologic and solid tumor treatment, low-dose MTX (LD-MTX, 5-25 mg weekly) has become the cornerstone therapy for rheumatoid arthritis due to its efficacy and generally favorable tolerability [1]. 

Despite its widespread use and safety profile, MTX can cause serious organ toxicity, including myelosuppression, mucositis, hepatotoxicity, and kidney injury, particularly when drug interactions or changes in renal function occur [2]. Among the most dangerous drug interactions is the combination of MTX with trimethoprim-sulfamethoxazole (TMP-SMX), a commonly prescribed bacteriostatic antimicrobial. Both medications inhibit folate metabolism, and TMP-SMX additionally impairs renal excretion of MTX, creating a synergistic effect that can result in a mean 66% increase in systemic MTX exposure [3].

Importantly, while therapeutic drug monitoring (TDM) protocols are well-established for HD-MTX therapy, no such monitoring protocols exist for LD-MTX. Serum MTX levels in LD-MTX toxicity correlate poorly with clinical severity, as the pharmacokinetics are highly variable and unpredictable [4].

Presented is a fatal case of MTX toxicity in an elderly patient with multiple comorbidities who developed severe pancytopenia and hemorrhagic oral mucositis following concurrent administration of TMP-SMX for cellulitis. Crucially, this severe clinical toxicity occurred despite a measured serum MTX concentration of 0.07 μmol/L, well within the non-toxic range. This case highlights the lethal potential of this well-documented drug interaction and demonstrates that clinical presentation, rather than drug levels, must guide management decisions in suspected LD-MTX toxicity.

## Case presentation

**Figure 1:**
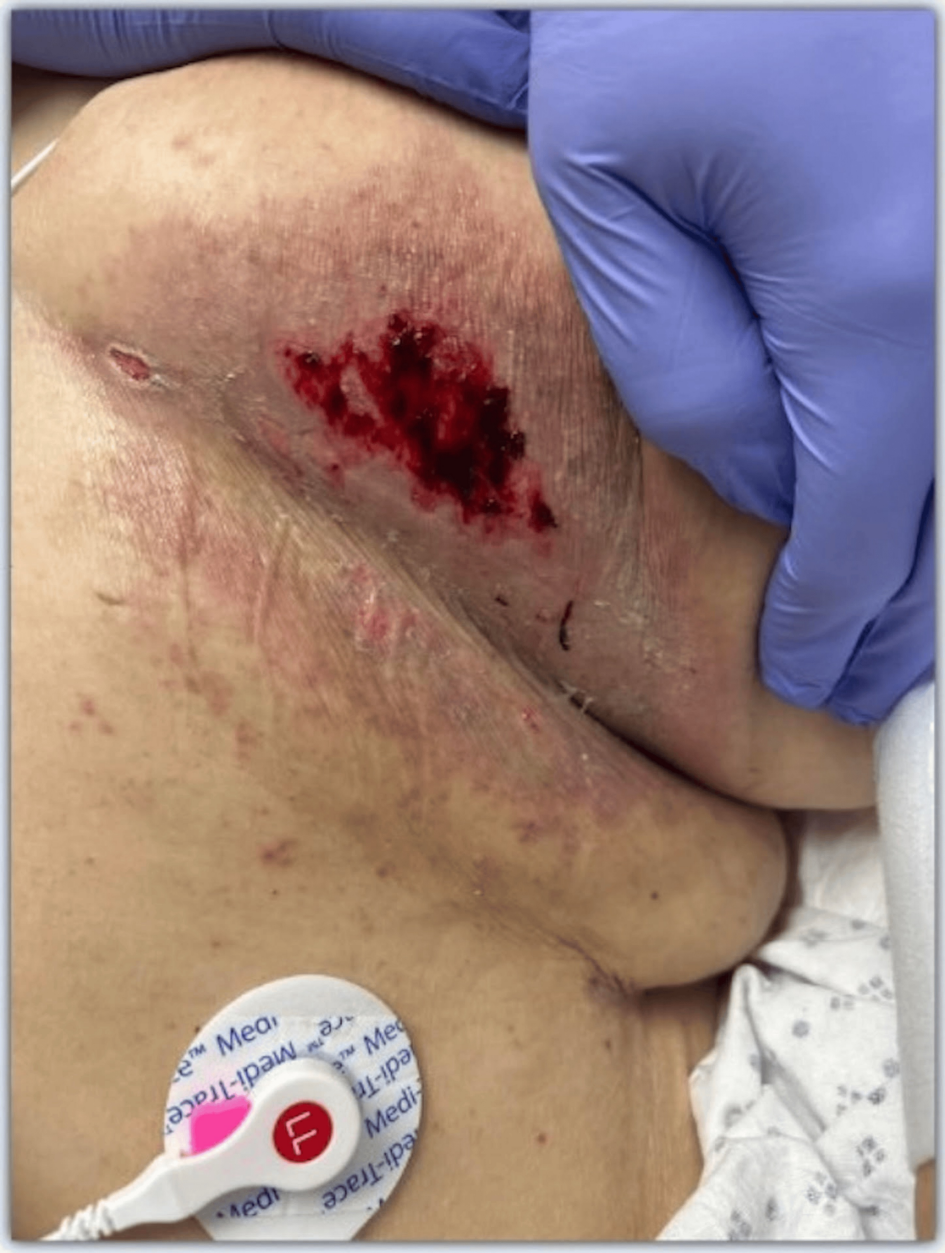
Under-breast hemorrhagic bullae

The patient was a 69-year-old female with a significant past medical history including osteoarthritis, rheumatoid arthritis, psoriatic arthritis, chronic hypoxic respiratory failure secondary to interstitial lung disease, type 2 diabetes mellitus, heart failure with preserved ejection fraction, hypertension, and hyperlipidemia. She presented to the emergency department (ED) from a skilled nursing facility with chief complaints of shortness of breath, an under-breast rash (Figure 1), and right lower extremity cellulitis. Her home medication regimen for her rheumatologic conditions included MTX 20 mg weekly and folic acid 1 mg daily. She reported receiving her weekly MTX dose 14 days prior to the ED visit. Crucially, she had also completed a five-day course of double-strength (DS) trimethoprim/sulfamethoxazole (Bactrim) orally twice daily (BID) 10 days before admission for her right lower extremity cellulitis.

Upon ED evaluation, laboratory work-up was significant for new onset pancytopenia and an elevated creatinine (Table 1). The patient was febrile on day 1 of admission, with a maximum temperature of 101 degrees Fahrenheit. Shortness of breath was initially attributed to anemia. Imaging studies ruled out common etiologies of acute respiratory decompensation: an ultrasound duplex excluded deep vein thrombosis in the bilateral lower extremities, and a computed tomography angiography of the chest showed no pulmonary embolism but did reveal peribronchial opacities bilaterally, concerning for an infectious or inflammatory process. The patient was started on vancomycin and Zosyn. Blood cultures remained negative throughout the admission. 

**Table 1:** Laboratory values during hospitalization This table summarizes the most severe point (nadir or peak) reached for critical laboratory parameters during the course of hospitalization.

Laboratory test	Unit of measurement	Nadir / peak value	Day of occurrence (relative to admission)	Normal reference range (approximate)
Hematology				
White blood cell count (WBC)	k/ul	0.41	Day 4	4.5-11.0
Absolute neutrophil count (ANC)	k/ul	0.08	Day 4	1.8-7.70
Platelets	k/ul	24	Day 4	150-450
Hemoglobin (Hgb)	g/dL	6.2	Day 0	12-17
D dimer	ng/ml	2842 (Peak)	Day 0	<500
Haptoglobin	mg/dl	340 (Peak)	Day 0	63-273
Iron	ug/dl	93	Day 0	50-170
Reticulocyte Production Index	RPI	0.3	Day 0	>2
LDH	u/L	411	Day 0	125-220
Vitamin B12	pg/ml	198	Day 0	213-816
Folate	ng/ml	3.4	Day 0	>9
PTT	seconds	48.1 (Peak)	Day 3	24-36
PT	seconds	17.5 (Peak)	Day 3	10-13
INR		1.5 (Peak)	Day 3	0.8-1.2
Renal function				
Serum creatinine	mg/dL	1.47 (Peak)	Day 0	0.6-1.2 mg/dL
Blood urea nitrogen (BUN)	mg/dL	35 (Peak)	Day 0	7-20 mg/dL
Hepatic function				
Aspartate aminotransferase (AST)	U/L	36 (Peak)	Day 0	10-40
Alanine aminotransferase (ALT)	U/L	24 (Peak)	Day 0	7-56
Total bilirubin	mg/dL	0.5 (Peak)	Day 0	0.3-1.2 mg/dL
Methotrexate level				
MTX Level (random, 16 days later)	umol/l	0.07	Day 2	<0. 1

**Figure 2:**
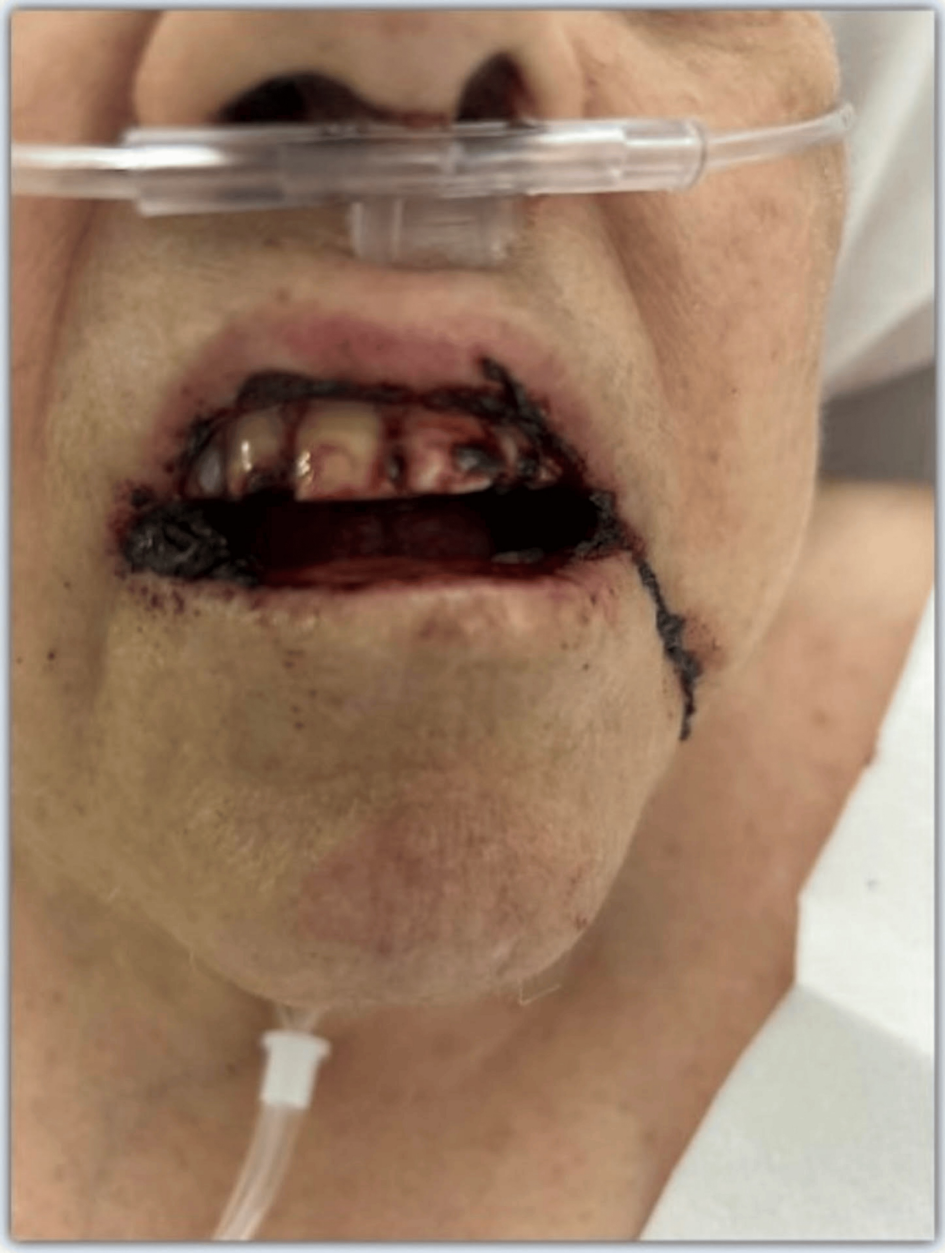
Oral mucositis

On day 2, a regional hematologist was consulted to address the new onset of pancytopenia. The hematologist's opinion was that the clinical picture was consistent with neutropenic fever, and it was recommended to continue broad-spectrum antibiotics and to correct cytopenias as indicated. A random MTX level collected on day 2 of admission was 0.07 umol/l (Table 1). The patient began complaining of mouth pain on day 1 of admission, progressing to overt bleeding documented on day 4 (Figure 2). Her clinical course rapidly deteriorated on the night of day 4 with confusion and worsening oral mucocutaneous hemorrhage, with the inability to protect her airway. 

The patient was in a Do Not Resuscitate (DNR) status upon admission. Following a comprehensive family discussion, the patient's daughter upheld the DNR decision. Given the severe mucocutaneous hemorrhage and impending respiratory arrest, the decision was made to transition the patient to comfort care. The patient passed away on day 5 of admission.

## Discussion

LD-MTX generally demonstrates a favorable safety profile. However, life-threatening toxicity can occur and manifest across multiple organ systems: myelosuppression ranging from isolated cytopenias to profound pancytopenia, mucocutaneous toxicity presenting as oral mucositis and gastrointestinal ulceration, hepatotoxicity, and nephrotoxicity [2,5]. Our patient, with pre-existing risk factors including advanced age, renal impairment, and folate deficiency, developed profound pancytopenia (Table 1), hemorrhagic oral mucositis (Figure 2), and neutropenic fever following a short course of TMP-SMX. The following sections further explain the mechanisms of this interaction, the limitations of serum MTX levels, the missed opportunity for leucovorin rescue, and why empiric folic acid supplementation failed to protect this patient.

Mechanisms of drug interaction

The interaction between MTX and TMP-SMX is multifactorial and particularly dangerous due to synergistic mechanisms that amplify toxicity. Both medications interfere with folate metabolism through complementary pathways: MTX competitively inhibits dihydrofolate reductase (DHFR), preventing the conversion of dihydrofolate to tetrahydrofolate, while trimethoprim also inhibits DHFR, and sulfamethoxazole blocks an earlier step by inhibiting dihydropteroate synthase. This creates an additive antifolate effect that severely impairs DNA synthesis in rapidly proliferating tissues, particularly bone marrow and gastrointestinal mucosa [3,6].

TMP-SMX also interferes with MTX clearance through multiple pharmacokinetic mechanisms. The sulfonamide component competitively inhibits renal tubular secretion of MTX, leading to elevated and prolonged plasma concentrations. Studies have demonstrated a significant decrease in renal clearance of free MTX (from 12.1±6.8 to 5.6±2.4 ml/kg/min) when co-administered with TMP-SMX. In addition, displacement from plasma protein binding sites increases the free fraction of MTX (from 37.4±11% to 52.2±6.4%), further enhancing toxicity potential. These combined changes result in a mean 66% increase in systemic exposure to MTX [3,6].

Therapeutic drug monitoring: high-dose vs. low-dose MTX

A critical distinction exists between the utility of TDM in HD versus LD MTX therapy. In HD-MTX oncology protocols, standardized monitoring is integral to safe administration. Serum MTX concentrations are measured at defined intervals-typically at 24, 48, and 72 hours post-infusion-with established thresholds guiding clinical decision-making. Delayed clearance is generally defined as MTX levels ≥10 μmol/L at 24 hours, ≥1 μmol/L at 48 hours, or ≥0.2 μmol/L at 72 hours, and these values directly inform leucovorin rescue dosing and duration. This pharmacokinetic monitoring has been the foundation of HD-MTX safety protocols for over 30 years [7].

In stark contrast, no such validated monitoring protocols exist for LD-MTX toxicity. TDM is not recommended in the LD setting because the pharmacokinetics of MTX at these doses are highly variable and unpredictable, and no standard threshold concentrations have been established to predict toxicity [8]. In our patient, the serum MTX level of 0.07 μmol/L-collected on Day 2 of admission, approximately 16 days after her last MTX dose and 12 days after completing the TMP-SMX course, was well below the commonly cited toxic threshold of 0.1 μmol/L. However, this level provided false reassurance. In LD-MTX toxicity complicated by drug-drug interactions, serum concentrations correlate poorly with clinical severity, particularly when measured days after the inciting exposure. The MTX level reflects only circulating drug at a single point in time and does not account for the cumulative cellular damage that has already occurred from intracellular MTX polyglutamates, which can persist in tissues long after plasma levels have normalized [7,8].

Failure of clinical recognition: the absent leucovorin rescue

A sobering aspect of this case is that leucovorin rescue was never initiated, nor does documentation suggest it was considered. The regional hematologist who was consulted on Day 2 to address the pancytopenia characterized the clinical picture as consistent with sepsis and neutropenic fever, recommending continuing broad-spectrum antibiotics and correcting cytopenias as indicated. Notably, MTX toxicity was not mentioned as a potential etiology despite the documented recent TMP-SMX exposure in a patient on chronic MTX therapy. It is plausible that the treating clinicians did consider MTX toxicity but were falsely reassured by the non-toxic serum MTX concentration, leading them to pursue alternative explanations for the patient's deterioration.

This represents a critical gap in recognition. Leucovorin (folinic acid) rescue remains the cornerstone of management for severe MTX toxicity. By providing reduced folates that bypass the MTX-induced blockade of DHFR, leucovorin can restore cellular function. However, timing is critical-tissue toxicity may become irreversible if leucovorin therapy is delayed beyond 40-48 hours from the onset of toxicity [9,10]. Current evidence supports intravenous leucovorin at doses of 15-25 mg every 6 hours for severe LD-MTX toxicity, though even with appropriate treatment, mortality remains substantial at 30-40% [11]. In our patient, by the time severe mucocutaneous hemorrhage manifested on Day 4, the window for effective leucovorin rescue had likely passed.

Failure of empiric folic acid supplementation

While folic acid supplementation is the standard of care for patients receiving weekly MTX therapy, shown to reduce toxicity scores, discontinuation rates, and hepatotoxicity without compromising efficacy [12], this protective measure did not prevent catastrophic toxicity in our patient. Her serum folate level at admission was 3.4 ng/ml (normal >9 ng/ml), indicating significant folate deficiency despite presumably being prescribed folic acid supplementation. Additionally, her vitamin B12 level was low at 198 pg/ml (normal 213-816 pg/ml).

This finding underscores several important points. First, folic acid supplementation is only protective if patients are adherent, and medication compliance in elderly patients with multiple comorbidities residing in skilled nursing facilities may be inconsistent. Second, even with adequate supplementation, the additive antifolate effects of MTX combined with TMP-SMX can overwhelm the protective capacity of folic acid. Third, pre-existing folate depletion creates particular vulnerability to the combined antifolate effects of both drugs. Low baseline folate and vitamin B12 levels have been identified as risk factors predictive of future MTX toxicity [12]. In this case, the patient's depleted folate stores meant she had minimal reserves to buffer the synergistic antifolate assault of concurrent MTX and TMP-SMX therapy.

## Conclusions

The MTX-TMP-SMX interaction represents a preventable cause of life-threatening toxicity. Despite extensive documentation in the literature, including case reports, observational studies, and systematic reviews, this interaction continues to occur, highlighting critical gaps in clinician education and system-based preventive measures.

This case demonstrates several key lessons. First, even a single short course of TMP-SMX can precipitate fatal toxicity in patients on stable LD-MTX therapy. Second, serum MTX levels should not be used to rule out LD-MTX toxicity; the constellation of pancytopenia, mucositis, and documented drug interaction should prompt immediate leucovorin rescue regardless of MTX concentration. Third, folic acid supplementation, while beneficial, does not provide absolute protection, particularly in the setting of drug interactions or underlying folate deficiency.

Prevention must be the primary focus, as mortality remains substantial even with appropriate treatment. Clinicians must recognize that clinical presentation, not MTX levels, should guide the decision for immediate aggressive intervention in suspected cases.
